# From Metal-Related Public Health Risks to Bioremediation: The Potential of the Polyextremophilic *Galdieria* spp.—A Systematic Review

**DOI:** 10.3390/ijms27156855

**Published:** 2026-07-30

**Authors:** Elio Pozzuoli, Concetta Auciello, Salvatore Avilia, Manuela Iovinella, Mario De Stefano, Sabrina Esposito, Stefania Papa, Claudia Ciniglia

**Affiliations:** 1 Department of Environmental, Biological and Pharmaceutical Sciences and Technologies, University of Campania “Luigi Vanvitelli”, Via Vivaldi 43, 81100 Caserta, Italy; elio.pozzuoli@unicampania.it (E.P.); concetta.auciello@unicampania.it (C.A.); salvatore.avilia@unicampania.it (S.A.); manuela.iovinella@unicampania.it (M.I.); mario.destefano@unicampania.it (M.D.S.); sabrina.esposito@unicampania.it (S.E.); stefania.papa@unicampania.it (S.P.); 2 Department of Biology, University of York, Wentworth Way, York YO10 5DD, UK

**Keywords:** *Galdieria*, bioremediation, biosorption, metal recovery, critical raw materials, circular economy, health, horizontal gene transfer

## Abstract

The growing demand for rare earth elements (REEs), heavy metals (HMs) and precious metals (PMs) has intensified interest in sustainable recovery strategies from secondary resources, including mining residues, industrial effluents and waste electrical and electronic equipment (WEEE). These streams represent exposure interfaces, because soluble and bioavailable metal species may persist, bioaccumulate and contribute to oxidative stress, genotoxicity, carcinogenic outcomes and chronic systemic effects. This systematic review, conducted following PRISMA guidelines, evaluates the thermoacidophilic red microalga *Galdieria* spp. as an extremophilic platform for metal bioremediation, recovery and upstream risk reduction. *Galdieria* spp. combines tolerance to low pH, elevated temperature and high metal loads with rapid surface biosorption and, in living biomass, slower intracellular sequestration and detoxification. Its interaction with REEs, PMs and toxic HMs is mediated by cell-wall functional groups, extracellular polymeric substances, redox-active processes and metabolic flexibility shaped partly by horizontal gene transfer (HGT). The review discusses matrix complexity and adsorption–desorption cycles, highlighting their implications for real industrial streams. Overall, *Galdieria* spp. emerges as a robust extremophilic bio-interface for selective metal recovery, hazardous waste mitigation, circular-economy biorefinery models and prevention of metal-associated risks to environmental and human health, while current scale-up limitations and process-oriented research priorities are identified.

## 1. Introduction

Technological transformations in recent decades have driven an unprecedented global demand for strategic metallic elements, including Rare Earth Elements (REEs), Precious Metals (PMs), and Heavy Metals (HMs). Expansion of electronics and renewable energy technologies has intensified the extraction and environmental dispersion of these elements [1]. Consequently, metals previously confined to geological deposits are gradually entering surface biogeochemical cycles through mining and industrial processes. In particular, waste electrical and electronic equipment (WEEE) has emerged as one of the fastest-growing waste categories globally. This massive accumulation highlights an urgent necessity to treat e-waste as a secondary source of strategic metals, while reducing environmental and human risks [2,3,4].

The environmental dispersion of these elements poses severe risks to ecosystems and human health, modulated heavily by bioavailability, solubility, and chemical speciation [5,6]. Heavy metals such as lead, cadmium, and arsenic are persistent, non-biodegradable toxins that bioaccumulate through food chains, causing cellular damage and physiological dysfunctions [7,8,9]. Concurrently, REEs can interfere with various cellular processes, inducing oxidative stress and metabolic alterations across diverse organisms [10]. Because long-term or occupational exposure to these metal mixtures presents a pressing public health concern, developing upstream removal strategies serves as a vital preventive measure to mitigate contamination before environmental discharge [10,11,12,13]. However, conventional hydrometallurgical and engineered separation methods face significant limitations when treating chemically aggressive secondary resources. Industrial effluents and electronic waste leachates are typically characterized by extremely low pH, high temperatures, and high ionic strength, which undermine standard mesophilic biological systems [14]. To overcome these operational barriers, attention has turned to extremophilic microorganisms capable of operating under chemically aggressive conditions. Among them, the thermoacidophilic red microalga of the genus *Galdieria* Merola, 1982 has emerged as a promising platform for metal biorecovery [15,16,17,18,19]. This systematic review aims to critically evaluate the potential of *Galdieria* spp. for the bioremediation and selective recovery of REEs, PMs, and HMs from acidic and chemically complex secondary streams. The first part establishes a comprehensive framework detailing the sources, anthropogenic mobilization pathways, and severe ecotoxicological and public health risks associated with the accumulation of REEs, PMs, and HMs in surface biogeochemical cycles. Building upon this assessment of environmental urgency, the second part of the review delivers a critical analysis of the genus *Galdieria* as a sustainable platform for the selective recovery and bioremediation of these strategic metals from complex secondary streams. Synthesizing insights from geochemistry, microbial ecology, and process engineering, we examine the precise biochemical mechanisms that govern metal–algal interactions under extreme conditions [20,21]. Furthermore, the paper evaluates the operational flexibility of using living versus inactivated biomass to decouple metal capture from growth constraints [18]. Ultimately, this work outlines integrated process chains where *Galdieria* serves as a high-efficiency unit operation for upstream bioremediation, successfully bridging resource valorisation with long-term environmental protection [22].

## 2. Methods

The present review was conducted following PRISMA guidelines [23]. A literature search was performed using bibliographic databases (Scopus, Web of Science, PubMed) and a full-text platform (ScienceDirect) as a supplementary source for article retrieval.

**Search strategy and time frame:** The present study considers articles and reviews written in English and published from 1 January 2015 to 21 July 2026. Primary experimental studies constituted the direct systematic evidence base, whereas review articles were used as secondary sources to support the broader contextual and mechanistic discussion and to identify additional primary studies through citation searching. The search strategy was designed to capture two complementary bodies of evidence: the initial strand of research focuses on the interactions between *Galdieria* and metals (core stream); the second strand of research focuses on the health and toxicological aspects relevant to the metal classes discussed (context stream).

**Core stream (metal–*Galdieria* interactions):** In Scopus, the following query was applied to titles, abstracts, and keywords: TITLE-ABS-KEY (*Galdieria* AND (metal OR “rare earth elements” OR lanthanum OR yttrium OR “precious metal” OR Au OR Ag OR Pt OR Pd OR Rh OR biosorption OR bioaccumulation OR biomining OR bioremediation)) AND (LIMIT-TO (LANGUAGE, “English”)). Equivalent queries were adapted for Web of Science and PubMed using the corresponding field tags.

Web of Science was searched using: ALL = (*Galdieria* AND (metal OR “rare earth” OR lanthanum OR yttrium OR “precious metal” OR Au OR Ag OR Pt OR Pd OR Rh OR biosorption OR bioaccumulation OR biomining OR bioremediation)), with filters for English and the period 2015–2026.

PubMed was searched using: (*Galdieria* AND (metal OR “rare earth elements” OR lanthanides OR yttrium OR gold OR silver OR platinum OR palladium OR biosorption OR bioaccumulation)), with filters for English and the period 2015–2026.

**Context stream** (health and exposure relevance): To support the dedicated health-focused discussion, an additional search was conducted without constraining the query to *Galdieria*, using terms related to exposure and toxicity of the metal classes addressed (e.g., “rare earth elements” OR lanthanides OR “heavy metals” OR “platinum group metals” OR Au OR Ag OR Mo OR molybdenum OR molybdate) combined with health-related keywords (e.g., toxicity, genotoxicity, carcinogenicity, exposure, occupational, e-waste, WEEE, leachate). During revision, a supplementary targeted search combining “*Galdieria*” with “molybdenum” or “molybdate” was also performed. This search did not identify studies directly addressing Mo uptake, biosorption, recovery, or bioremediation by *Galdieria* spp.

**Eligibility criteria:** Original peer-reviewed primary studies reporting empirical data relevant to metal removal, metal–microalga interactions, physiological or molecular responses, bioremediation, biosorption, bioaccumulation, or metal recovery were eligible for the direct systematic evidence synthesis. Review articles were retained as secondary sources to support the broader contextual and mechanistic discussion and to identify additional primary studies through citation searching; they were not treated as independent experimental observations. The original research preprint and selected institutional or grey-literature sources were used only for contextual purposes and were not included in the direct systematic evidence synthesis. Primary studies were excluded when they focused on biological systems or processes outside the predefined scope, were not written in English, or lacked sufficient empirical or methodological information to interpret their findings.

**Figure 1:**
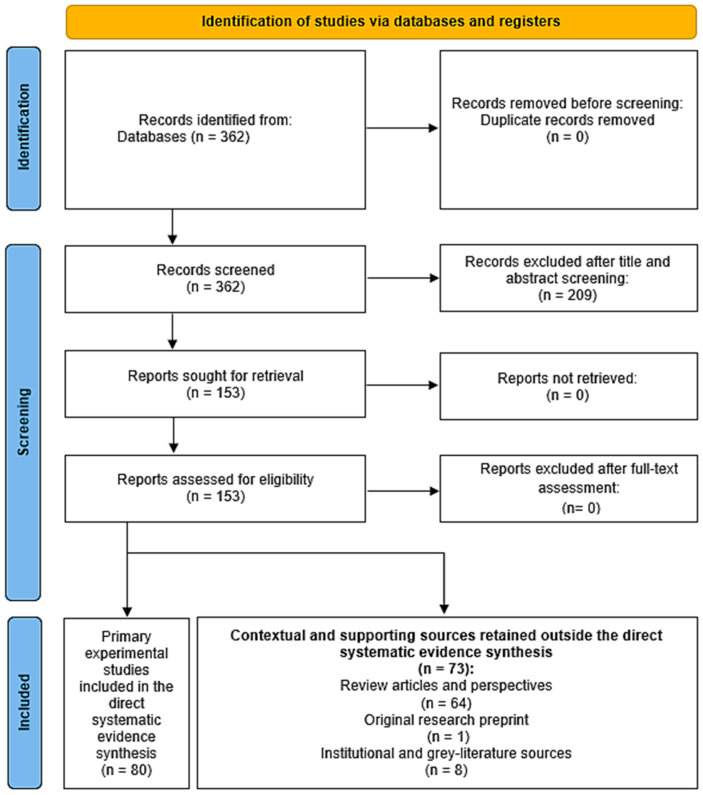
PRISMA flow diagram [24].

**Screening and study selection:** Two reviewers independently conducted the first stage of screening (title and abstract). A third reviewer assessed eligibility in cases of doubt. Articles meeting inclusion criteria were retrieved in full text and re-evaluated. Duplicate records were removed prior to screening. During full-text assessment, publications were classified into three categories: primary experimental studies eligible for the direct systematic evidence synthesis; secondary or contextual sources, including review articles, the original research preprint, and selected institutional sources; and reports excluded after full-text assessment. Secondary sources were retained for contextual discussion and citation searching but were not treated as independent experimental observations. Full-text exclusions and their reasons were recorded separately in the PRISMA flow diagram (Figure 1).

**Data extraction and synthesis**: The following information was extracted when available: algal strain and biological system; metal characteristics and speciation; cultivation and exposure conditions; mechanisms of interaction and molecular responses (including EPS and cell-wall involvement); HGT-related traits; metal removal performance; process feasibility and applications; economic and sustainability considerations. Given the heterogeneity of study designs and reporting formats, a meta-analysis was not performed and findings were synthesised narratively, organised into thematic categories aligned with the review framework.

**Risk of bias**: A formal risk-of-bias assessment (e.g., ROBIS or GRADE) was not applied due to heterogeneity; however, each study was assessed qualitatively based on reporting transparency, completeness of methods and outcomes, and declared conflicts of interest.

## 3. Results and Discussion

### 3.1. Sources and Biological Implications of Strategic Metals

**Figure 2:**
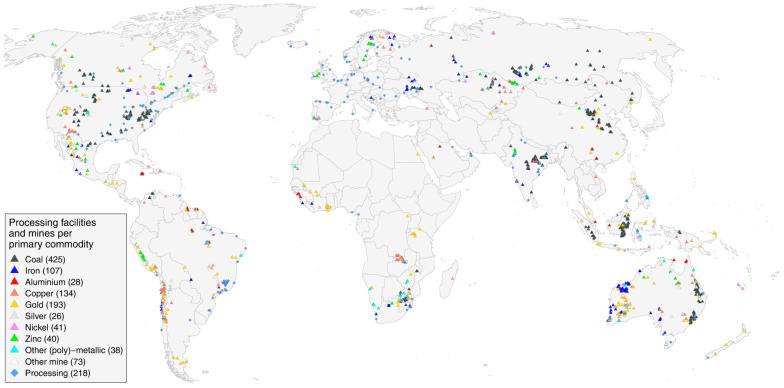
Worldwide distribution of mines and processing facilities that drive the increasing mobilization of metals in modern industrial systems [26].

Technological transformations in recent decades have led to the increased movement of many metallic elements that play a key role in modern industrial systems. REEs, PMs, and HMs are chemically distinct, yet they all have growing environmental relevance. Increased demand for high-performance materials, expansion of the electronics sector, and development of technologies for the energy transition have intensified the extraction, use and environmental dispersion of these elements [1]. This anthropogenic mobilisation extends beyond primary extraction, as processing residues, industrial discharges, and end-of-life technological products increasingly redistribute metals into soils, sediments, and aquatic systems [1]. Mining and processing facilities for major primary commodities are widely distributed across all continents, reflecting the large spatial footprint of extractive activities and the growing anthropogenic mobilization of strategically relevant metals (Figure 2).

The rapid proliferation of electronic devices and their shortening life cycles have made WEEE one of the fastest-growing waste streams worldwide, already exceeding 50 million tonnes annually, according to the Global E-waste Monitor. Accordingly, WEEE represents both a concentrated reservoir of recoverable elements and a relevant source of environmental exposure when metals are released during informal disposal, leaching, or recycling processes [2,3,4,25].

#### 3.1.1. Rare Earth Elements

Rare earth elements are valued for their unique electronic and magnetic properties, which make them indispensable for a wide range of technological applications, especially in the manufacture of high-performance permanent magnets, electronic devices, industrial catalysts, and innovative energy systems. REEs are known as “industrial vitamins” due to their role in the development of high-efficiency technologies [10]. Despite their name, REEs are not actually that rare. However, it is rare to find deposits with sufficiently high concentrations, making extraction and separation processes complex and costly. These processes require intensive chemical treatments, which can generate effluents capable of contaminating surrounding ecosystems [1]. It is therefore necessary to intercept secondary sources of REEs such as industrial wastewater (IWW), atmospheric emissions, and electronic waste streams, which contain significant amounts of these elements [4]. The growing environmental presence of REEs has sparked scientific interest in their potential impact on living organisms. Recent studies have demonstrated the ability of these elements to interfere with various cellular processes, such as redox mechanisms and the activity of antioxidant enzymes, leading to oxidative stress and metabolic alterations in a variety of organisms [10]. Field studies have also documented REE accumulation in marine red, brown, and green algae and seagrasses, supporting their use as bioindicators and potential biosorbents [27]. These observations suggest that REEs may be involved in more complex biological interactions than previously recognised, while their behaviour in acidic solutions is strongly influenced by metal speciation and competition for binding sites—two factors that directly affect the design of *Galdieria*-based systems [10,28,29].

#### 3.1.2. Precious Metals

Precious metals (PMs), including Au, Ag, and platinum group metals (Pt, Pd, Rh), are strategic elements widely used in electronics, catalysts, and energy technologies. Their release is mainly driven by human activities, particularly electronic waste, which has increased interest in recovery from secondary sources [2,3]. PGMs have also been reported in urban and industrial soils, sediments, and aquatic systems, with potential ecotoxicological implications [17]. In *Galdieria*-based systems, their relevance lies in their distinct chemistry under strongly acidic, chloride-rich conditions, which differs from that of cationic metals and affects binding and transformation processes [30].

#### 3.1.3. Heavy Metals

The main anthropogenic sources of HMs include mining, industrial processes, the combustion of fossil fuels, and the disposal of electronic waste, which is a significant source of environmental contamination [3,4]. HMs, including lead (Pb), cadmium (Cd), mercury (Hg), chromium (Cr), and arsenic (As), have been extensively studied due to their toxicity, their ability to bioaccumulate in living organisms, and their persistence in the environment. This phenomenon can be attributed to the fact that a significant percentage of electronic components contain toxic metals that can be released into the environment during disposal or recycling processes. There is a large body of literature highlighting the toxicity of HMs. One of the main mechanisms of toxicity is their ability to interfere with cellular proteins and enzymes, thereby inducing alterations in fundamental metabolic processes and promoting the formation of reactive oxygen species (ROS), causing cellular damage, genetic alterations, and physiological dysfunctions in a variety of organisms, including humans [10]. Technology-intensive manufacturing streams, including wastewater from the integrated-circuit industry, can also contain complex mixtures of recoverable and toxic metals, creating a dual need for pollution control and resource recovery [31]. Molybdenum should be considered separately from the broad and chemically heterogeneous category of heavy metals. Although essential at trace concentrations, Mo may be released into aquatic and terrestrial environments through mining, mineral processing, and other industrial activities. Its occurrence in tailings pore waters, mine drainage, and waters affected by industrial use, together with the influence of pH, redox conditions, and chemical speciation on its environmental mobility, supports its explicit consideration in environmental exposure assessments [32].

### 3.2. Impacts of Metals on Human Health

The potential effects of metals on human health depend on exposure route, absorption, and subsequent toxicity, which are determined by physical and chemical characteristics, concentration, size, and solubility. The toxicity of soluble metals differs from that of insoluble metals, and soluble metals are generally more toxic due to higher bioavailability. Solubility is influenced by the extraction solvent and the conditions under which extraction occurs. In the context of secondary sources and metal-rich waste streams, these parameters become critical because pH and speciation can simultaneously modulate bioavailability and determine the feasibility of removal technologies [5,6].

#### 3.2.1. Rare Earth Element Exposure and Toxicity

Human exposure to REEs is primarily associated with anthropogenic activities. Occupational exposure is a major concern for workers in the mining, refining, and e-waste recycling sectors. Environmental exposure may occur in proximity to mining areas, via the food chain, and through the utilisation of gadolinium-based contrast agents in magnetic resonance imaging procedures. Lifestyle factors such as smoking and alcohol consumption may increase exposure levels [11,33]. REE bioaccumulation has been reported in animals, cereals, and leafy vegetables cultivated near mining areas [34,35,36,37]. Tobacco smoking is a significant source of these elements, with higher levels of La, Ce, Eu, Nd, Gd, and Yb reported in biological matrices such as semen and milk in long-term and passive smokers [38]. As reported by Rim [10], high traffic volume represents an additional source due to the use of cerium oxide nanoparticles as a diesel fuel additive. Until recently, research on REEs as xenobiotics has been limited, and the available evidence on acute toxicity and long-term bioaccumulation is inconclusive [10,11,39]. The available toxicological literature is uneven across elements, with greater coverage for Ce, La, and Gd. REEs can enter the human body through inhalation, ingestion, and skin contact [11], and elevated levels have been reported in urine, hair, and blood of individuals working in mining and e-waste recycling and in populations living near mines [40,41]. These elements can be localised in multiple cellular compartments and can persist in the body over extended periods. Mechanistic studies indicate multifactorial toxicity, including altered gene expression linked to oxidative stress [10,42]. Evidence supports ROS generation, redox imbalance, lipid peroxidation, oxidative DNA damage, micronuclei formation, chromosomal aberrations, and modulation of enzymes involved in oxidative stress, senescence, survival, proliferation, and apoptosis [42,43]. Additional mechanisms include altered telomerase activity and cell-cycle distribution [11] with potential links to tumour growth, neurodegeneration, and ageing [42,44]. Due to similarities in ionic radius, REEs may interfere with Ca-dependent regulation, with consequences for mitochondrial membrane potential, ATP production, and apoptosis [10]. Health effects associated with REE exposure include effects on bone mineral density and toxicity affecting the liver, lungs, blood, and nervous system, anaemia, and endomyocardial fibrosis [45,46]. Other reported implications include gastrointestinal symptoms, fatigue, hypertension, altered serum proteins, disruption of potassium channels, and changes in immunoglobulin levels [11,47]. Diesel particulate matter containing cerium oxide and ferrocene nanoparticles, as well as neodymium magnets, have been associated with reduced viability of human lung epithelial cells [48]. Chlorides of Ce, La, Eu, and Yb are cytotoxic to human embryonic kidney cells [49]. Elevated levels of multiple elements, including HMs and REEs, have been reported in brain tumour patients [50], and concentrations of La, Ce, and Ga have been reported as higher in blood and tumour tissue than in controls [50,51], although causal interpretation remains limited [52].

Several factors influence the chemical reactivity of REEs, including pH and oxic/anoxic conditions, and properties differ between nanoparticulate and ionic forms; oxidation state may vary across subcellular compartments. Long-term epidemiological evidence remains limited and contradictory, reflecting variability by geography, lifestyle, and occupational context as well as dose–response behaviour at higher exposure levels [42,43]. Consequently, REEs should be considered a plausible emerging risk class for human health, particularly in occupational and technology-driven exposure settings where concentrations are expected to increase over time [10,11].

#### 3.2.2. Precious Metal Exposure and Toxicity

PMs are economically important and widely used in catalysts, jewellery, electronics, and medical equipment. Silver exhibits high electrical and thermal conductivity and antibacterial properties [53]. However, silver nanoparticles (Ag-NPs), widely used as antibacterial agents, increase human and animal exposure [54]. Silver ions, Ag compounds, and Ag-NPs can be toxic and have been categorised as highly toxic contaminants [12]. In vitro and in vivo studies have shown adverse effects of Ag-NPs [53,54]. Mechanistically, Ag-NP cytotoxicity has been described by a “Trojan horse” process in which particles are taken up and then ionised intracellularly, releasing Ag^+^ ions. Toxicity is associated with ROS generation and membrane and DNA damage, with genotoxicity reported in human lymphocyte cells and embryonic neural stem cells [47,54]. Exposure has been associated with increased Bax expression and TUNEL-positive cells (apoptosis) and increased LDH release (membrane disruption, necrosis) [47]. Smaller particles (<10 nm) are generally more toxic than larger nanoparticles, with toxicity modulated by surface charge, stabilising agents, and exposure duration and dose [55].

In the context of platinum group metals, dermal and respiratory exposure remain significant in specific occupational settings. It has been demonstrated that platinum, ruthenium, and palladium nanoparticles can permeate intact human skin. Exposure to soluble platinum salts has been demonstrated to correlate with urinary platinum excretion and respiratory sensitisation, asthma, allergic contact dermatitis, and other skin effects in refinery workers [56,57,58,59]. Gold is ubiquitous in the human environment because of its wide range of applications; exposure occurs through personal, domestic, and occupational contact with jewellery, dental devices, implants, therapeutic products, diagnostic procedures, and the food chain [60]. The toxicological risks associated with gold are generally low and are mainly linked to allergic contact reactions; by contrast, other metals such as Ni, Cr and Cu present in gold alloys exhibit more serious clinical problems, as discussed below.

The existing literature on the occupational health implications of several PMs is both limited and inconsistent. This situation indicates a clear need for further toxicological studies. In the context of technology-driven waste streams, including electronic waste and associated recycling processes, the metals that are instrumental in determining economic value can also serve as critical indicators of exposure relevance. Consequently, treatment strategies that effectively reduce soluble or bioavailable forms can play a pivotal role in mitigating risk while facilitating resource recovery.

#### 3.2.3. Heavy Metal and Molybdenum Exposure and Toxicity

Industrialisation, agriculture, and urbanisation contribute to the release of HMs into the environment, contaminating air, soil, and water [61,62]. Some HMs (e.g., Zn, Ni, Fe, Cu) are essential trace elements required for biochemical and cellular functions and for metalloenzymes and metalloproteins; however, exceeding critical limits can cause serious health problems, whereas others (e.g., Pb, Cd) are highly toxic even at low concentrations [7]. HMs are persistent and non-biodegradable and can bioaccumulate through food chains [8,9]. Human exposure occurs through contact, inhalation, and ingestion, and metals can accumulate in tissues over time; for example, cadmium has a biological half-life of 15–20 years. Health effects range from acute to chronic, depending on exposure level. Regulatory limits have therefore been established, including maximum contaminant limits by the US Environmental Protection Agency [63], and several HMs are included among priority toxic substances by the Agency for Toxic Substances and Disease Registry [64].

The toxicity of HMs depends on their oxidation state and chemical form. Arsenic occurs in multiple oxidation states and the trivalent As(III) and pentavalent As(V) forms are environmentally most relevant; inorganic As(III) is generally more toxic than As(V) [13]. Chromium occurs in oxidation states from +2 to +6, with Cr(III) and Cr(VI) being most common; Cr(VI) is more toxic due to high solubility, mobility, and oxidising capacity [9,13]. Mercury in aquatic environments is particularly hazardous because Hg(II) can be converted into methylmercury (MeHg), a potent neurotoxin [13]. At the cellular level, many HMs bind thiol groups, disrupt protein function, and impair antioxidant defences. Mitochondrial dysfunction and disruption of the respiratory chain promote ROS generation and reduce ATP synthesis, contributing to oxidative stress and damage to proteins and DNA. These processes can lead to mutations, altered transcription, epigenetic effects, and broader redox imbalance [43,65,66]. Epidemiological and mechanistic evidence supports links between HM exposure and carcinogenic outcomes; Cd, Ni, As(III), and Cr(VI) are classified as Group 1 carcinogens by the International Agency for Research on Cancer [67,68]. HMs can also modulate tumour suppressor pathways and metallothionein expression [50]. Cadmium exposure has been associated with increased risks of breast, lung, and pancreatic cancer [65], and can stimulate proliferation and angiogenesis through VEGF-related mechanisms [69]. Respiratory nickel exposure has been associated with lung fibrosis and lung cancer [6]. HMs can also affect the nervous system, particularly when species cross the blood–brain barrier, as reported for Pb^2+^ and MeHg, contributing to neurotoxicity and neurological outcomes [70]. Prolonged exposure above acceptable limits has been associated with systemic effects, including kidney, liver, lung, gastrointestinal, cardiovascular, immune, and reproductive disturbances, as well as dermatological effects such as contact dermatitis after exposure to nickel and cobalt [6,71]. Taken together, the health impacts of HMs are governed by chemical form, persistence, and exposure context; therefore, upstream removal from industrial effluents and secondary-source leachates can represent a preventive measure where mixtures and long-term low-dose exposure are plausible.

Molybdenum (Mo) requires separate consideration because it should not be indiscriminately included within the broad heavy-metal category. Mo is an essential trace element required for the activity of several molybdenum-dependent enzymes; however, exposure to excessive concentrations may produce adverse health effects. According to the Agency for Toxic Substances and Disease Registry, the most relevant toxicological concerns include respiratory effects following inhalation exposure to molybdenum oxides and renal effects following excessive exposure. Decreased body weight, anaemia and alterations in male reproductive parameters have also been observed, primarily in experimental studies, whereas the available evidence remains insufficient to establish several other effects conclusively in humans. The toxicological relevance of Mo therefore depends on its chemical form, exposure route, concentration and duration, supporting its explicit consideration in assessments of occupational and environmental metal exposure [32].

In general, widespread industrial use of metals and subsequent release into the environment poses risks to human health due to the scale of exposed populations and workers, and due to bioaccumulation across aquatic food chains [43]. Metal-rich industrial wastewater streams are therefore relevant exposure interfaces, and removal before discharge is a preventive measure when mixtures and bioavailable forms occur [11,13,72]. Within this framework, the following sections focus on the need for biological remediation technologies and removal strategies under chemically aggressive conditions, highlighting how extremophilic red microalgae of the genus *Galdieria* can support metal recovery while contributing to exposure reduction in secondary-source scenarios [10,11,12,13].

### 3.3. Biological Recovery of Metals: Implications and Prospects

The collective presence of REEs, PMs and HMs demonstrates how contemporary technological endeavours are progressively altering the global biogeochemical cycles of metals. As demonstrated by the global distribution of mining and processing facilities (Figure 2), the intensification of mining, the expansion of industrial systems and the accumulation of technological waste are increasing the mobilisation of these elements within natural ecosystems with environmental and human health consequences.

In this context, biological systems are not only subject to the toxic effects of metals, but also play a role in the transformation and immobilisation of metallic elements. Microorganisms and microalgae possess a variety of biochemical strategies that enable interaction with metal ions, including biosorption, bioaccumulation, and biomineralisation [73]. Consequently, the integration of knowledge from geochemistry, microbial ecology and biotechnology suggests that these organisms could play a pivotal role in developing sustainable strategies for the recovery of strategic metals from complex matrices, such as IWW and electronic waste. More broadly, microalgal platforms have been proposed for simultaneous wastewater remediation and co-product generation, including carotenoids, biofuels, and energy-rich biomass, as well as for the removal of pharmaceutical contaminants [74,75,76]. Among green microalgae, *Chlamydomonas reinhardtii* represents both a well-established model organism and a versatile biotechnological platform. Its extensive genetic resources, mutant collections, and molecular-engineering tools have supported the investigation and optimisation of processes relevant to wastewater treatment, nutrient recovery, pollutant removal, and biomass valorisation. Beyond remediation, *Chlamydomonas* biomass has been investigated for the production of biofuels, hydrogen, pigments, recombinant proteins, and other high-value products, illustrating the potential integration of wastewater treatment with biorefinery-based approaches. Recent experimental evidence has also demonstrated that living and dried *C. reinhardtii* biomass can adsorb metals such as Pb(II), Cr(III), and Cd(II) from mono-metal and multi-metal solutions. Accordingly, *Chlamydomonas* provides an important green-microalgal benchmark for combined bioremediation and bioproduct production. Compared with this genetically tractable model, *Galdieria* offers distinct operational advantages in highly acidic, warm, and metal-rich matrices; therefore, the two genera should be considered complementary platforms rather than directly competing alternatives [77,78].

In the context of this review, these effects highlight the importance of recovery systems such as *Galdieria* that remain functional under acidic and mixed-metal conditions, where upstream removal can contribute to both resource valorisation and exposure mitigation. These characteristics make *Galdieria* spp. particularly suitable for metal recovery from chemically aggressive industrial streams [14]. Bioremediation represents the conceptual and operational link between metal recovery and human-health protection. Metal-rich wastewaters, acidic leachates, mining residues and WEEE-derived streams should not be considered only as secondary sources of valuable elements, but also as potential exposure interfaces where metals may occur in soluble, mobile or bioavailable forms. Biological systems capable of binding, immobilising or accumulating metals can therefore contribute to two complementary goals: the recovery of strategically relevant elements and the reduction in environmental dispersion before discharge. This dual function is particularly relevant for extremophilic microalgae, because acidic and chemically complex matrices often limit the application of conventional biological systems. Within this framework, *Galdieria*-based processes may be interpreted not only as metal-recovery tools, but also as upstream bioremediation strategies aimed at lowering the potential transfer of toxic or potentially carcinogenic metals from industrial residues to environmental and human exposure pathways [22]. Red extremophilic microalgae of the genus *Galdieria* spp. are increasingly considered highly effective biological systems for interacting with a wide range of metal species, including REEs, PMs, and toxic HMs, under physicochemical conditions that usually inhibit most microorganisms [15,16,79,80,81]. Their ability to function at low pH, high temperature, and under high metal loads places this genus in an interesting position between biomining, bioremediation, and resource recovery technologies [82].

### 3.4. Galdieria spp. Features and Usefulness

The genus *Galdieria* comprises thermoacidophilic unicellular red microalgae belonging to the class Cyanidiophyceae (Rhodophyta), whose ecology is intrinsically linked to environments characterised by extreme physical and chemical conditions, including low pH, high temperatures and high concentrations of dissolved metals. In nature, these species occur in acidic geothermal habitats such as hot springs and volcanic soils. In these environments, the pH can fall to approximately 0–1 and temperatures can exceed 50–55 °C. These extreme conditions promote metal solubilisation and therefore expose the organisms to persistently high concentrations of metal ions [83,84]. The combination of extreme acidity and high metal loads in such environments represents a dominant selective pressure, severely limiting biodiversity and promoting organisms with highly specialised metal tolerance and management strategies [85]. Thermophilic microalgae are increasingly recognised as robust biotechnological systems for industrial applications, particularly in processes operating at elevated temperatures and under chemically aggressive conditions, where mesophilic systems often exhibit limited stability and performance. Their intrinsic enzymatic thermostability, metabolic flexibility, and tolerance of physicochemical stressors provide distinct operational advantages in high-load, high-temperature and acidic WW treatment frameworks [14,86,87]. Accordingly, the persistence of *Galdieria* spp. in acidic, metal-rich niches indicates that metal tolerance and interaction are integral components of its ecology rather than incidental traits. This is also clearly demonstrated by studies in complex anthropic systems in which *Galdieria* spp. has been successfully cultivated in IWW and digestates characterised by high chemical complexity [88,89].

In addition, recent biorefinery-oriented studies have explored the use of microalgae to treat oil-produced water. These studies have highlighted the potential of algal systems to operate under highly saline and chemically complex industrial conditions, while enabling the simultaneous recovery of resources and the valorisation of biomass. These findings further support the broader applicability of extremophilic microalgae in IWW management frameworks [90]. Among the various industrial matrices investigated, *Galdieria* spp. has demonstrated its capacity to thrive in swine wastewater and leachates derived from anaerobic digestion and thermal hydrolysis processes. These environments are characterised by the presence of nutrients, recalcitrant organic substances, salts and metals, which foster sustained productivity even under acidic conditions [88,91,92]. The use of pre-treatment processes such as catalytic ozonation of leachates has been shown to improve the cultivability of the algae [92,93]. This underscores the adaptability of *Galdieria* spp. not only to extreme natural environments, but also to anthropogenic matrices characterised by high chemical pressure, which closely resemble the conditions of its original habitats. The ability of *Galdieria* spp. to thrive in metal-rich environments is closely linked to metal immobilisation through a combination of physicochemical and biological mechanisms. Several studies indicate that metal removal is often dominated by a fast biosorption step at the cell surface that does not depend on metabolism, while in living cells this is followed by slower intracellular sequestration phenomena [89,94]. This difference between a rapid surface-dominated phase and a slower intracellular phase is also useful in practical terms, because it helps in choosing between living and inactivated biomass and in defining the operating window according to pH and metal speciation. More specifically, *Galdieria* spp. has shown very high efficiency in the recovery of PMs under strongly acidic conditions [17]. Ju et al. [89] observed a selective removal above 80–90% of Au(III) and Pd(II) within approximately 30 min using low biomass concentrations (about 1.4 mg mL^−1^ dry weight), even in solutions with up to 0.4–0.5 M HCl. This behaviour is not common among biological sorbents, whose efficiency usually declines substantially below pH 2, and it highlights once again how relevant surface chemistry is under extreme acidity [17,80,89].

These findings indicate that cell-surface chemistry is a key determinant of metal selectivity and recovery efficiency under extremely acidic conditions [18,19].

In addition to surface biosorption, *Galdieria*-mediated metal recovery can involve redox transformations that alter metal oxidation states and favour the formation of less soluble or elemental products. In the specific case of gold, adsorption is accompanied by progressive reduction from Au(III) to Au(I) and to Au(0), resulting in the formation of metallic nanoparticles and an effective increase in available binding sites via an autocatalytic mechanism [18]. These redox-driven processes have been described in microalgae as adaptive responses to metal stress [95] and support the interpretation that *Galdieria* spp. can behave as a chemically active system rather than a passive sorbent. Beyond noble-metal transformations, members of the Cyanidiophyceae have also demonstrated the capacity to mediate anaerobic Fe(II) bio-oxidation, indirectly enhancing As(III) removal through coupled redox pathways [96]. Together, these observations support a mechanistic continuum between surface coordination, redox transformation and process-relevant selectivity under extreme chemical conditions [18,95,96,97].

The efficiency and selectivity of metal removal can also change depending on how the biomass is treated. For example, freeze-dried *Galdieria* spp. biomass appears to maintain greater structural stability under strongly acidic conditions, and this can also improve the accessibility of surface functional groups [98]. Platinum recovery efficiencies above 90% have been reported even from multi-metal solutions, together with the possibility of carrying out repeated adsorption–desorption cycles without a clear loss of performance. This finding provides a practical design lever, because inactivated biomass can decouple metal capture from growth constraints while preserving performance in harsh matrices [18].

*Galdieria* spp. can also be applied to highly complex solid–liquid matrices. Biomass has been shown to biosorb REEs from fluorescent lamp phosphors and bauxite-derived suspensions, even in the presence of high concentrations of competing elements such as aluminium and iron. In phosphor leachates, yttrium displayed preferential adsorption, reaching up to 34.6 mg g^−1^ dry biomass within a short period [94]. In bauxite suspensions, overall recovery yields of 5–30% (*w*/*w*) have been reported using biologically assisted adsorption–desorption schemes [99]. These outcomes are notable when compared with non-extremophilic biosorbents such as commercial baker’s yeast, which has been shown to selectively recover Au(III) from strongly acidic aqua regia leachates of waste printed circuit boards, although near-complete recovery required a relatively high biomass loading and a three-stage batch operation [100]. Accordingly, comparisons across platforms suggest that performance differences arise not only from “biosorbent capacity,” but from the ability to function within acidity-speciation regimes that undermine less specialised systems.

### 3.5. Interactions Between Galdieria spp. And Metals: Mechanistic and Functional Characteristics

In *Galdieria* spp., metal tolerance and binding capacity seem to be more general features of the genus, suggesting the presence of conserved physiological and structural traits rather than isolated species-specific properties. The interaction with metals includes both rapid biosorption phenomena at the cell surface and slower intracellular mechanisms such as bioaccumulation, sequestration, and detoxification. The extent to which each of these processes contributes depends strongly on several factors, including pH, metal speciation, matrix complexity, and the physiological state of the biomass, i.e., whether it is living or non-living [20]. Additional information has also come from electrochemical methods such as stripping voltammetry, which have helped to better characterise the speciation and stability of the metal species associated with the algal surface in acidophilic systems. Taken together, these findings support the view that surface complexation is the predominant first step in metal interaction [21]. To ensure comparability across metal classes, the evidence is organised by metal group for clarity, but interpreted through a shared mechanistic framework: (i) pH and speciation define the dominant aqueous forms and competition regimes, (ii) cell-interface chemistry and EPS define the initial coordination mode, (iii) redox-active pathways may transform selected metals, and (iv) biomass status determines the feasibility of intracellular contributions and regeneration strategies [20,21]. For clarity, the evidence is discussed by metal class. However, the interpretation used throughout this section is intentionally cross-cutting: metal speciation defines the chemical starting point, cell-interface chemistry defines the first level of interaction, intracellular processes determine longer-term handling, and biomass state constrains the operational configuration. This shared structure allows direct comparison among metal classes that are often discussed separately in the literature. Accordingly, key speciation anchors, common cross-study pitfalls, and primary design levers across metal classes are summarised in Table 1 [20,21]. A critical limitation that is often overlooked in the current literature is the “misalignment between pH and speciation”. While many studies report optimal removal at specific pH values, they often fail to consider the dynamic shift in metal coordination caused by the biomass itself [101]. Future research should adopt standardised titration methods that integrate the protonation state of the cell surface with the speciation of metals in aqueous solution. This approach is preferable to simply reporting “final removal percentages”. This shift in perspective is essential to ensure data comparability across different laboratory systems and pilot plants.

#### 3.5.1. Rare Earth Elements and *Galdieria* spp.

**Speciation and pH window:** REEs are mainly present as trivalent cations (REE^3+^) in acidic aqueous environments, and their behaviour depends strongly on solution chemistry, since they can form both inorganic and organic complexes [28,29]. Several studies have shown that *Galdieria* spp. is able to remove REEs quite efficiently from acidic solutions derived from mining effluents, bauxite residue (red mud), and WEEE leachates, often without the need for extensive pretreatment steps [62,81,104].

Reported REE removal efficiencies are generally higher under moderately acidic conditions, approximately between pH 1.5 and 4.0, although the optimal range depends on metal speciation, matrix composition, and biomass state. At lower pH values, protonation of surface ligands and competition from H^+^ can reduce REE binding [62,94,98]. Therefore, REEs recovery by *Galdieria* spp. should be interpreted in relation to both aqueous metal speciation and the protonation state of cell-surface ligands, rather than pH alone.

**Binding mode at the cell interface:** Direct evidence is available for non-living *Galdieria* sulphuraria biomass. Manfredi et al. identified specific interactions between Ln^3+^ ions and cell-surface functional groups, showing that proteins and carbohydrates contribute differently depending on the lanthanide, and described the biosorption mechanism using a surface-complexation model [99]. These findings are consistent with broader evidence from bacteria and marine macroalgae, where REE uptake is mainly associated with negatively charged cell-wall and extracellular functional groups [29,105].

**Kinetics and mechanism partitioning:** Kinetic studies frequently demonstrate biphasic uptake behaviour. This is characterised by an initial rapid phase, which is dominated by physicochemical biosorption on the cell wall and EPS (frequently described by Langmuir or Freundlich models). This is followed by a slower phase in which intracellular bioaccumulation can become relevant, particularly after extended exposure in living cultures [62]. As demonstrated in the relevant literature, analogous patterns have been reported in other algal systems, such as *Gracilaria gracilis*, indicating a general algal feature expressed under more extreme conditions in *Galdieria* spp. [106].

**Operational mode and matrix complexity:** Recovery from solid and semi-solid matrices is challenging due to limited accessibility and heterogeneity, while the scale-up and environmental implications of biotechnological REE recovery from bauxite residue remain important constraints [107]. *Galdieria* spp. has been reported to recover REEs directly from bauxite powders via repeated adsorption–desorption cycles, enabling reuse of biomass and progressive enrichment [104,108]. Immobilisation strategies, including entrapment in calcium alginate beads, have enhanced operational stability and removal efficiency, supporting simultaneous REE recovery and nutrient removal from ammonium-rich wastewater [89]. Similar immobilised red algae configurations have demonstrated effective REE recovery directly from ammonium-rich wastewaters [37]. In addition, biosorption from fluorescent lamp phosphors and bauxite-derived suspensions has been reported even in the presence of competing aluminium and iron, with preferential yttrium adsorption reaching up to 34.6 mg g^−1^ dry biomass in phosphor leachates and overall yields of 5–30% (*w*/*w*) in bauxite suspensions using biologically assisted adsorption–desorption schemes [80,94,99].

#### 3.5.2. Precious Metals and *Galdieria* spp.

**Speciation, acidity, and chlorocomplex dominance:** Interactions with precious metals (Pt and Pd) typically occur as stable anionic chlorinated complexes (e.g., PtCl_6_^2−^, PdCl_4_^2−^) in acidic waste streams, while Au is commonly present as AuCl_4_^−^ in aqua regia and similar leachates [109]. Recovery is challenging due to electrostatic repulsion between negatively charged biological surfaces and anionic metal complexes. Despite these constraints, *Galdieria* spp. has shown high performance under extreme acidity. Sun et al. [110] reported removal efficiencies above 94–96% for PtCl_6_^2−^ from acidic solutions, with evidence of adsorption accompanied by transformation processes (reduction and/or precipitation) and partitioning between EPS/cell wall association and intracellular detection.

**Binding mode: inner-sphere coordination and ligand specificity:** Under strongly acidic conditions, PMs capture can involve ligand-specific inner-sphere interactions. Ju et al. [89] reported selective removal above 80–90% of Au(III) and Pd(II) within approximately 30 min using low biomass concentrations (approximately 1.4 mg mL^−1^ dry weight), even with 0.4–0.5 M HCl [17,89]. Spectroscopic and single-cell studies indicate that Au and Pd capture involves inner-sphere coordination with nitrogen- and sulfur-containing functional groups, with the prevailing binding mode being controlled by metal speciation and solution acidity [18,19].

**Redox-active capture and nanoparticle formation:** For gold, adsorption can be accompanied by progressive reduction from Au(III) to Au(I) and ultimately Au(0), resulting in metallic nanoparticle formation and an effective increase in available binding sites through an autocatalytic mechanism [18]. Such redox-driven processes have been described as adaptive responses to metal stress in microalgae [95] and support the interpretation that *Galdieria* spp. can function as a chemically active system rather than a passive sorbent. Beyond noble-metal transformations, Cyanidiophyceae members have demonstrated the capacity to mediate anaerobic Fe(II) bio-oxidation, indirectly enhancing As(III) removal through coupled redox pathways [96].

#### 3.5.3. Heavy Metals and *Galdieria* spp.

**Speciation and priority contaminants in acidic matrices:** *Galdieria* spp. show pronounced tolerance to divalent HMs such as Cd^2+^, Pb^2+^, Ni^2+^, and Zn^2+^, which are priority contaminants in acidic mine waters and industrial effluents. *Galdieria* spp. can sustain growth and metabolic activity at mg L^−1^ levels under acidic conditions [7,22,102]. In broader environmental contexts, electronic waste is also a relevant source of heavy-metal dispersion [2,4].

**Binding mode and biphasic behaviour:** Heavy-metal removal typically follows a biphasic pattern: a rapid biosorption phase driven by interactions with cell-wall functional groups and EPS, followed by slower intracellular processes that become increasingly significant over longer timescales. Competition for binding sites and transport pathways is evident in multi-metal systems, yet *Galdieria* spp. maintains appreciable removal efficiency in chemically complex conditions [7].

**Intracellular handling: chelation and detoxification:** A distinguishing feature is robust intracellular detoxification capacity. *Galdieria* spp. has been reported to maintain unusually high intracellular glutathione levels compared with mesophilic green microalgae such as Chlorella sorokiniana, including under sulphur-deprived conditions [111]. A thiol-rich intracellular environment has been proposed to support chelation and sequestration of HMs, reducing cytosolic toxicity and enabling sustained growth, consistent with stress-response mechanisms described under metal-induced oxidative pressure in other microalgal systems [103,111]. Metallothionein-like proteins are frequently implicated in intracellular metal binding and detoxification.

**Contact-time-dependent metal handling:** Heavy-metal management in *Galdieria* spp. highlights a practical distinction between rapid metal capture at the cell interface and longer-term biological handling. Short-contact configurations reflect surface-driven biosorption mediated by cell-wall and EPS functional groups, whereas prolonged exposures increasingly involve intracellular chelation, sequestration, and detoxification processes [7,103,111].

#### 3.5.4. Considerations and Perspectives

Several unifying principles emerge across REEs, PMs, and HMs. pH and metal speciation consistently act as dominant control variables influencing solubility, surface charge, and binding affinity. While extreme acidity promotes solubilisation, it also increases competition between protons and metal ions for surface binding sites [112]. In addition to physicochemical parameters, biomass physiological status can modulate uptake efficiency. For instance, nutrient limitation and ionic competition have been observed to affect transport systems and binding capacity, and potassium deprivation has been reported to enhance cation removal efficiency in other microalgal systems [113]. Non-living biomass offers several advantages, including its capacity to support reusable and regenerable biosorption systems, as well as its ability to enhance process control under toxic conditions [30].

On this basis, three main working hypotheses emerge. Firstly, under acidic conditions, the predominant metal species may offer a more reliable indication of the interaction mode than the metal category in isolation. Secondly, the equilibrium between surface coordination and intracellular processing may determine not only the efficiency of removal, but also the stability and regeneration potential under complex mixtures. Thirdly, inactivated biomass may be preferable in strongly acidic systems dominated by chlorocomplexes, where growth-dependent contributions become less relevant than interface reactivity and process robustness. These cross-metal differences are summarised in Table 2, which compares the predominant chemical forms, operational pH ranges, biomass states, and prevailing interaction mechanisms for REEs, PMs, and HMs.

These findings link metal-interaction mechanisms to biotechnological applications and environmental-health relevance. The next section considers how evolutionary adaptation, including HGT, may support *Galdieria* spp. performance under extreme conditions.

### 3.6. Horizontal Gene Transfer and Extreme Adaptation in Galdieria spp.

#### 3.6.1. Horizontal Gene Transfer in Microalgae

HGT has been identified as a fundamental process in the evolution of microeukaryotes, particularly within taxa that inhabit extreme environments. Several comparative genomics studies have revealed that protists have incorporated genes of bacterial or archaeal origin into their genomes, thereby obtaining functions related to ion transport, detoxification, the response to oxidative stress, and the utilisation of complex organic substrates [115,116]. Substantial genome-based classification initiatives further substantiate this hypothesis, demonstrating that numerous groups of microalgae and photosynthetic protists exhibit a significant share of horizontally acquired genes, which are often associated with ecologically relevant functions [117]. In this context, HGT appears to be a recurring mechanism that contributes to the formation of ‘mosaic’ genomes, particularly in environments with strong selective pressure.

#### 3.6.2. HGT-Driven Adaptation and Ecological Interactions in *Galdieria* spp.

***Galdieria* spp. as a model case of mosaic genome**: Within the broader context of HGT in microalgae, *Galdieria* spp. represents a well-documented example of an extremophilic red microalga whose genome has been substantially shaped by gene acquisition from bacterial and archaeal lineages. Genes of putative horizontal origin have been associated, primarily through comparative genomic and phylogenetic analyses, with chemical resistance, heterotrophic metabolism, membrane transport, metal and metalloid homeostasis, and oxidative-stress responses [116,118]. These functions may contribute to the ability of *Galdieria* spp. to survive under highly acidic, metal-rich, and thermally extreme conditions, although their individual physiological roles require functional validation. Gene exchange within geothermal microbial communities may also promote the circulation of adaptive traits among bacteria, archaea, and microeukaryotes, contributing to the ecological resilience of Cyanidiophyceae [119].

**Heavy metals, oxidative stress and resilience**: Metal exposure can increase ROS production and induce substantial physiological alterations in microalgae [120]. In *Galdieria* spp., redox enzymes, efflux pumps, and membrane transporters, some of which show phylogenetic affinities consistent with a possible prokaryotic origin, may contribute to metal tolerance and redox homeostasis. However, their specific roles under metal exposure have not yet been functionally demonstrated. The evolutionary relevance of gene acquisition is further supported by evidence of both vertical inheritance and HGT in arsenic-resistance pathways across eukaryotic lineages [121]. Whereas synthetic-biology approaches aim to introduce metal-tolerance and remediation traits into model organisms, *Galdieria* spp. possesses a genetic repertoire shaped by prolonged evolutionary selection under extreme environmental pressures [122].

**Algal symbiosis and emerging technologies**: Interactions between algae and associated bacteria may influence metal bioavailability through the production of chelating compounds, extracellular polymeric substances, and chemically transformative processes [123]. Algae–bacteria systems are increasingly investigated for wastewater treatment because their complementary metabolic capabilities support the removal of nutrients, metals, and persistent organic pollutants [73,124,125,126,127]. In this context, the metabolic versatility of *Galdieria* spp. may support the development of resilient algae–bacteria systems under acidic and chemically complex conditions. However, a direct contribution of HGT-derived functions to consortium-level performance has not yet been demonstrated.

**HGT contribution to large-scale microalgal diversification**: Viruses may also contribute to microalgal evolution by promoting genomic rearrangements, introducing genes, and modifying regulatory networks involved in environmental-stress responses [128]. In *Galdieria* spp., these large-scale evolutionary processes converge with bacterial and archaeal gene acquisition, contributing to a highly specialised but metabolically versatile mosaic genome [116,119].

#### 3.6.3. Functional Consequences of Horizontal Gene Transfer

Evidence framework and limits of inference. The molecular basis of metal tolerance and recovery in *Galdieria* spp. should be interpreted by distinguishing experimentally demonstrated phenotypes, transcriptional responses, phylogenetic evidence, and functional predictions. Within the broader evolutionary framework, HGT is recognised as an important contributor to the formation of mosaic genomes and to functional diversification in eukaryotes [116,119]. Experimental studies have demonstrated condition-dependent Fe bioaccumulation in living *G. sulphuraria*, as well as rapid REEs biosorption by freeze-dried whole-cell biomass and post-extraction cell debris. These findings confirm metal association, accumulation, and retention at the phenotypic level, but they do not identify the proteins responsible for these processes or establish a causal contribution of HGT-derived genes [129,130].

**Transporter-mediated metal homeostasis:** Available genomic and transcriptomic evidence suggests that membrane transport is an important component of metal homeostasis in *Galdieria* spp. Following short-term exposure of *G. yellowstonensis* SAG 107.79 to Ce, transcripts associated with the Gene Ontology categories “Localization” and “Transport” included members of the Major Facilitator Superfamily (MFS), Amino Acid–Polyamine–Organocation (APC), and natural resistance-associated macrophage protein (Nramp) families [118]. Phylogenetic analyses supported possible horizontal acquisition for selected MFS and APC transporters, whereas Nramp proteins appeared to be predominantly vertically inherited [118]. However, their substrate specificity, subcellular localisation, and direct contribution to metal tolerance or recovery have not yet been experimentally demonstrated.

Additional evidence for the involvement of ABC, ZIP, and cation diffusion facilitator (CDF) transporters in metal-stress responses is available from the closely related thermoacidophilic red microalga *Cyanidioschyzon merolae*. Under long-term Ni exposure, transcriptomic analyses revealed the downregulation of the ABC transporter genes *CmMRP1* and *CmATM/HMT-1* and of the CDF-family member *CmMTP2*, whereas the ZIP-family genes *CmZIP1* and *CmZIP2* were upregulated under the highest Ni concentration across the investigated time points [131]. These results provide experimental evidence that these transporter families respond transcriptionally to metal stress in an extremophilic red microalga. However, the study did not directly measure transporter activity or establish whether the observed transcriptional changes caused Ni tolerance or altered intracellular accumulation. Moreover, because these findings were obtained in *C. merolae*, the corresponding functions in *Galdieria* spp. remain to be experimentally validated.

Recent genetic evidence from the model green microalga *Chlamydomonas reinhardtii* further supports a potential involvement of ABC-type transporters in metal homeostasis. Through insertional mutagenesis and screening under different molybdate concentrations, Leon-Miranda et al. identified disruptions in genes encoding ABC-type transport proteins in both a Mo-resistant and a Mo-sensitive mutant. The Mo-resistant mutant carried an insertion in *Cre05.g234400*, encoding a predicted ABCG-family transporter, whereas the Mo-sensitive mutant was disrupted in *Cre03.g191350*, encoding a predicted ABCA-family transporter. Based on the contrasting phenotypes, the authors proposed that the former may participate in Mo uptake or intracellular storage, whereas the latter may contribute to Mo efflux. These findings provide genetic evidence linking ABC-type transport proteins to Mo homeostasis in a model microalga. However, direct molybdate-transport activity, protein localisation, and the proposed uptake or efflux functions remain to be validated through biochemical and targeted genetic analyses [132]. Although these results were obtained in a phylogenetically distant green microalga, they demonstrate how mutant-based functional screening can complement transcriptomic and comparative-genomic predictions. Similar approaches will be required to determine whether ABC transporters contribute directly to metal uptake, efflux, or intracellular sequestration in *Galdieria* spp.

**Chelation and antioxidant defence**: Following cellular uptake, metal toxicity may be mitigated through intracellular chelation and redox-control mechanisms. Metallothioneins are cysteine-rich, gene-encoded proteins, whereas phytochelatins are glutathione-derived peptides enzymatically synthesised in response to metal exposure. Both systems can bind metal ions and promote their sequestration or compartmentalisation. Metallothioneins, phytochelatins, glutathione, and intracellular storage compartments have been proposed as components of metal detoxification in microalgae [133]. Elevated intracellular glutathione levels have also been reported in *G. sulphuraria* [111], supporting the presence of a substantial thiol-based redox buffer. However, these observations were not obtained under controlled metal-specific exposure and therefore do not directly demonstrate a metal-detoxification pathway. In addition, their molecular identity, metal-dependent regulation, and functional contribution in *Galdieria* remain insufficiently characterised.

Antioxidant enzymes, including superoxide dismutase, catalase, peroxidases, and glutathione-dependent enzymes, may also contribute to the control of metal-induced ROS and maintenance of redox homeostasis. However, the available literature does not yet provide a comprehensive metal-specific characterisation of these enzymes in *Galdieria*. Their involvement should therefore be considered biologically plausible but not fully demonstrated. Future studies should determine enzyme activity, transcriptional regulation, and intracellular redox changes under controlled exposures to individual metals and realistic multi-metal mixtures.

**Transcriptomic and phylogenomic context**: The available transcriptomic evidence indicates that transport-related genes respond rapidly to metal exposure, but transcriptional induction alone does not demonstrate that a protein directly transports the metal or determines recovery performance. Similarly, phylogenetic affinity with bacterial, archaeal, or fungal homologues supports a possible evolutionary origin but does not establish the present physiological function of the acquired gene. Recent genome-scale analyses have also revealed at least seven well-supported *Galdieria* lineages, together with partially independent and incongruent evolutionary trajectories of nuclear, plastid, and mitochondrial genomes. Consequently, transporter repertoires, regulatory responses, and metal-interaction phenotypes may differ substantially among species and strains and should not be generalised across the genus without comparative validation [134].

**Functional interpretation and research priorities**: Current evidence supports the hypothesis that HGT contributed to the evolutionary diversification and metabolic flexibility of *Galdieria* spp. [116,118]. However, it does not yet demonstrate a direct causal relationship between individual HGT-derived genes and metal-recovery performance. Metal biosorption, bioaccumulation, transporter induction, and evolutionary origin should consequently be treated as related but distinct levels of evidence. Functional validation through targeted gene disruption, heterologous expression, protein localisation, transporter assays, time-resolved transcriptomics, proteomics, and subcellular metal quantification is required to determine which transport, chelation, antioxidant, and sequestration pathways directly control metal tolerance and recovery.

#### 3.6.4. Comparison with Other Metal Recovery Strategies

Metal recovery from secondary sources usually relies on integrated process chains rather than single-step solutions, particularly for mining wastewaters in which treatment and resource recovery must be considered together [135]. Hydrometallurgical, pyrometallurgical, and bio-hydrometallurgical routes can efficiently mobilise or concentrate metals, but they often generate streams that still require selective downstream chemical separation. In this context, biological platforms are most useful as selective unit operations, especially under acidic and high-ionic-strength conditions [109]. Table 3 summarises the main recovery routes and the most plausible integration points for *Galdieria*-based processes.

Recent advances in engineered sorbents, including ion-exchange resins, functionalised polymers, metal–organic frameworks, and composite materials, have shown high selectivity for metal recovery from complex matrices [136]. Other promising approaches include bio-templated materials, modified lignocellulosic sorbents, ion-imprinted polymers, and red mud–carbon composites, although their performance mainly depends on structural design and surface chemistry [137,138,139,140,141].

Against this background, *Galdieria* spp. is distinctive because its performance derives from biological adaptation rather than only material design. Its tolerance and metal-interaction capacity may reflect, among other factors, an evolutionary history that includes putative HGT events. However, a direct causal relationship between these events and process-level metal-recovery performance has not yet been demonstrated. Unlike engineered sorbents, its recovery performance may be modulated through biomass state, stress conditioning, and process-window selection. Overall, *Galdieria* spp. emerges as a flexible biological platform for selective metal recovery from acidic and chemically complex waste streams.

**Table 3:** High-level comparison of major recovery routes from secondary sources and plausible integration points for *Galdieria*-based selective capture, pre-concentration, and polishing under acidic conditions.

Route	Typical Role in Recovery Chains	Main Strengths	Main Limitations	Most Plausible Integration Point for *Galdieria*	References
Hydrometallurgy (acid leaching; chloride media)	Dissolution and mobilisation of metals into leachates; downstream separation required	High extraction efficiency; broad applicability to secondary sources; compatible with multi-step separations	Chemically aggressive leachates (low pH, high ionic strength, complex speciation); downstream selectivity becomes limiting	Selective capture/polishing from acidic leachates; pre-concentration where speciation and pH compromise conventional biosorbents	[109]
Pyrometallurgy (thermal treatment/smelting)	Thermal concentration into recoverable phases; often combined with subsequent leaching	Industrial maturity for some streams; rapid throughput; concentrates metals	High energy demand; off-gas management; limited selectivity without additional steps; downstream hydrometallurgy often required	Downstream after leaching of pyrometallurgical products: *Galdieria* can act in acidic polishing streams where selectivity is required	[142]
Bio-hydrometallurgy (bioleaching)	Biologically mediated mobilisation of metals into solution (leachate generation)	Potentially reduced chemical intensity in selected contexts; can act on low-grade feeds	Slower kinetics; sensitivity to matrix constraints; produces leachates that still require selective separation	Downstream selective capture from bioleachates; complements mobilisation with selective recovery/polishing	[82,142]
Biological capture (biosorption/bioaccumulation; incl. *Galdieria*)	Selective binding/uptake from aqueous streams; can be applied as unit operation	Operable in acidic matrices using *Galdieria*; adaptable modes (living vs. non-living); regeneration possible	Requires process design around speciation, kinetics, and regeneration; performance depends on matrix complexity	Unit operation for selective capture and pre-concentration in integrated chains; particularly relevant for acidic, metal-rich leachates and multi-metal systems	[4,143]

## 4. Future Perspectives

**Process scale-up and techno-economic validation:** The transition of *Galdieria*-based metal recovery from laboratory experiments to industrial implementation will require the development of reactor configurations adapted to acidic, saline, and multi-metal streams. Living cultures may be integrated into suspended, attached-growth, membrane, or immobilised-cell photobioreactors, whereas non-living biomass and post-extraction cell debris may be more suitable for packed-bed, fluidised-bed, or cartridge-based adsorption systems. Reactor selection should account for mass-transfer limitations, hydraulic residence time, light availability, mixing requirements, fouling, and competition among dissolved ions. Pilot-scale algal wastewater-treatment studies have also shown that seasonality, operating temperature, and hydraulic conditions can affect treatment performance and strain selection [144]. The feasibility of operating *Galdieria*-based systems beyond bench scale is supported by the POWER platform developed for municipal wastewater treatment. This system combined outdoor cultivation of *G. sulphuraria* in closed photobioreactors, simulated continuous operation, contaminant removal, biomass production, and downstream hydrothermal liquefaction. The reviewed configuration reached an operational batch volume of approximately 700 L and highlighted biomass harvesting, drying, energy demand, and reactor footprint as major scale-up constraints [145]. Although the POWER platform was not specifically designed for selective metal recovery, it provides an important process-level benchmark for the cultivation, hydraulic management, biomass valorisation, and scale-up of *Galdieria*-based technologies. Experience from broader algal biofuel and carbon-capture systems further indicates that reactor choice, harvesting, co-product valorisation, and residual biomass use strongly influence scale-up feasibility and process economics [146,147].

Biomass harvesting also remains a major operational constraint because centrifugation and filtration can substantially increase energy demand and process costs. Immobilisation, flocculation, sedimentation, magnetic functionalisation, and the reuse of residual biomass after bioproduct extraction may reduce downstream processing requirements. An integrated biorefinery approach, in which high-value C-phycocyanin is recovered before residual biomass is used for metal capture, may improve overall process economics and material efficiency [130,148]. Recent studies involving post-extraction *Galdieria* debris and *Galdieria*–ZIF-8 composites illustrate possible routes for integrating biomass valorisation, enhanced adsorption, material recovery, and biosorbent reuse [130,149,150]. However, adsorption capacity alone is insufficient to establish practical feasibility. Regeneration efficiency, eluent consumption, metal recovery from concentrated eluates, loss of biosorbent performance over repeated cycles, biomass replacement, waste generation, and corrosion-resistant equipment should be quantified under continuous-flow conditions. Pilot-scale studies using real industrial streams, together with techno-economic analysis and life-cycle assessment, are therefore required to compare *Galdieria*-based systems with ion exchange, chemical precipitation, solvent extraction, and conventional biosorbents.

**Molecular engineering and digital optimisation: Future** improvements may arise from the integration of comparative genomics, transcriptomics, proteomics, metabolomics, and ionomics to identify strains combining metal tolerance, uptake capacity, selectivity, growth rate, and suitable biomass properties. The substantial genomic divergence detected among *Galdieria* lineages indicates that omics-guided strain selection should precede the generalisation of metal-interaction phenotypes across the genus [134]. Transcriptomic responses and comparative transporter analyses may then support the identification of candidate genes involved in metal uptake, efflux, intracellular compartmentalisation, thiol-mediated chelation, and oxidative-stress control [118].

Within this framework, molybdenum represents a particularly relevant but unexplored target, as no direct studies have yet evaluated Mo uptake, biosorption, or recovery by *Galdieria* spp. Comparative evidence linking ABC-type transporters to Mo homeostasis in *Chlamydomonas reinhardtii* provides candidate mechanisms that could guide future functional investigations in *Galdieria* [132].

Synthetic biology and metabolic engineering could subsequently be applied to candidate transporters, chelation pathways, antioxidant systems, cell-wall components, and extracellular polymeric substances. Although these approaches have not yet been functionally established for metal recovery in *Galdieria*, nuclear gene targeting and stable transgene expression have been demonstrated in the related thermoacidophilic red microalga *C. merolae*. These advances include a two-step nuclear gene-targeting method and, more recently, a modular transformation system enabling heterologous gene expression and metabolic engineering [151,152].

CRISPR–Cas genome editing may provide a future route for validating candidate genes and generating strains with modified metal tolerance or selectivity. DNA-free CRISPR–Cas9 editing has already been demonstrated in other microalgae, including Chlamydomonas reinhardtii [153], but the available literature does not yet document a validated CRISPR platform for *Galdieria*. Its application to this genus should therefore be presented as a future research direction rather than an established capability. Genome engineering should also consider possible trade-offs among metal accumulation, cellular fitness, biomass productivity, and process stability.

In parallel, machine-learning approaches could support data-driven strain screening and process optimisation. A recent study developed predictive models for heavy-metal adsorption by algae-based biosorbents using variables related to biomass properties and operational conditions, including biomass dose, initial metal concentration, pH, contact time, and temperature [154]. Machine-learning models have also been used to predict microalgal harvesting efficiency by magnetic nanoparticles, illustrating their potential for reducing experimental screening requirements [155]. However, these approaches still require validation with *Galdieria*-based systems under continuous-flow, multi-metal, and pilot-scale conditions.

## 5. Conclusions

Human activities have intensified the mobilization of rare earth elements, precious metals and toxic heavy metals into aquatic and terrestrial ecosystems, increasing the persistence of these contaminants and amplifying their risks for environmental and human health. Since these elements are non-biodegradable and can interfere with redox homeostasis, protein function and genomic stability, effective upstream treatment strategies are essential to reduce their entry into broader biogeochemical pathways.

In this context, *Galdieria* emerges as a promising biological platform for both bioremediation and resource recovery. Its thermoacidophilic lifestyle gives it exceptional tolerance to high acidity, elevated temperature and metal-rich matrices, making it particularly suitable for conditions in which conventional biological sorbents often lose efficiency or structural integrity.

Metal interaction with *Galdieria* can be interpreted as a biphasic process. The first phase consists of rapid, metabolism-independent biosorption mediated by functional groups located on the cell wall and extracellular polymeric substances. This is followed by a slower intracellular phase involving active transport, chelation and detoxification mechanisms in living cells. This dual behaviour explains why both inactivated biomass and viable cultures can be useful, depending on the intended application.

From an operational perspective, the possibility of using living cultures or inactive biomass gives *Galdieria*-based systems substantial flexibility. Inactivated biomass may be preferable when rapid metal capture, robustness and process simplicity are required, whereas living cultures may be more suitable when intracellular accumulation, biological selectivity or long-term adaptation to complex waste streams are targeted.

*Galdieria* spp. should therefore be considered not only as a bioremediation agent, but also as a selective pre-concentration unit within integrated industrial treatment chains. Its application upstream of conventional recovery or polishing steps could support the valorisation of secondary resources, including electronic waste leachates, mining residues and acidic industrial effluents, while simultaneously reducing the mobility and bioavailability of toxic metals.

Future development of this platform will require a transition from descriptive biosorption studies to more mechanistic and predictive approaches. Genome-informed strain selection, validation of metal-specific transport and chelation pathways, and pilot-scale testing under continuous-flow conditions with realistic multi-metal matrices will be essential to assess scalability and reproducibility.

Despite the promising results reported to date, the available evidence remains difficult to compare because studies differ considerably in pH, metal speciation, ionic strength, matrix composition, biomass state, contact time, and analytical approaches. Consequently, apparently contradictory removal efficiencies may reflect methodological heterogeneity rather than genuine biological differences. Major research gaps include the limited use of real multi-metal industrial streams, the insufficient distinction between biosorption, bioaccumulation, reduction, and precipitation, the scarcity of regeneration and long-term cycling studies, and the lack of continuous-flow, pilot-scale, techno-economic, and life-cycle assessments. A further specific gap is the absence of direct experimental studies on Mo uptake, biosorption, and recovery by *Galdieria* spp., particularly under the acidic and chemically complex conditions characteristic of mining residues and industrial effluents. The role of algae-bacteria consortia also remains largely unexplored, particularly regarding their potential to enhance extracellular polymeric substance production, metal transformation, process resilience, and selectivity under acidic conditions. Future studies should therefore integrate standardised chemical characterisation, mechanistic and multi-omics approaches, controlled consortia experiments, and direct benchmarking against conventional and engineered recovery systems.

Overall, *Galdieria*-based technologies provide a conceptual and operational bridge between environmental protection and circular resource recovery. By combining metal detoxification with the recovery of critical and precious elements, this microalgal platform offers a sustainable route for linking industrial bioprocessing, ecosystem protection and long-term human health preservation.

## Figures and Tables

**Table 1:** Speciation anchors, cross-study pitfalls, and primary design levers for interpreting *Galdieria*–metal interactions under acidic conditions.

Metal Class	Speciation Framework	Main Cross-Study Pitfall	Primary Process Lever	Key Evaluation Parameter	References
REEs	Dominant REE form (REE^3+^ vs. complexed species), ionic strength, competing cations	pH values not comparable across studies; speciation/competition not reported	Define and control the pH window; report speciation and competition explicitly	Selectivity under multi-metal competition; pH-dependent capacity	[21,34,85,89,90]
PMs	Chlorocomplex dominance (Cl^−^ level), acidity, redox conditions	Comparisons without chloride/acidity; biomass state differs (living vs. freeze-dried)	Select biomass state and regeneration strategy; operate within chlorocomplex regimes	Kinetics at pH ≤ 2 and regenerability over cycles	[20,22,23,24,82,83,91,92]
HMs	Free cations versus complexes; mixture composition and toxicity pressure	Contact time not comparable; growth effects confounded with sorption	Contact-time design (short vs. long) and growth constraints	Interface capture and intracellular handling separation	[7,22,102,103]

**Table 2:** Concise overview of the predominant chemical forms, operational pH ranges, biomass states, and prevailing interaction mechanisms across the different metal classes.

Metal Class	Representative Metals	Predominant Chemical Form in Solution	Operational pH Range	Biomass State	Dominant Interaction Mechanism	Key Remarks and Applications	References
REEs	La, Y, Eu, Nd, Sm	REE^3+^; inorganic complexes (e.g., carbonate, phosphate)	~1.5–4.0 (study-dependent)	Living and non-living	Surface biosorption (cell wall, EPS) with partial intracellular bioaccumulation	High efficiency in acidic leachates; suitable for pre-concentration from mining residues, bauxite, red mud, and WEEE	[62,81,94,98,104]
PMs	Pt, Pd, Au	Chlorinated anionic complexes (PtCl_6_^2−^, PdCl_4_^2−^); AuCl_4_^−^ (aqua regia)	≤2.0; down to ~1.0 for Au in strongly acidic chloride media	Living and non-living (preferentially freeze-dried)	EPS-mediated adsorption and inner-sphere complexation (N/S ligands), coupled with chemical reduction and/or precipitation	Effective removal of high-value metals under extreme acidity; biomass reuse enables recovery from metallurgical and refining waste streams	[17,18,19,89,109,110,114]
HMs	Cd, Pb, Ni, Zn	Divalent cations (Cd^2+^, Pb^2+^, Ni^2+^, Zn^2+^)	~1.5–4.0 (study-dependent)	Living (predominant)	Rapid biosorption followed by intracellular chelation and detoxification	High tolerance and sustained growth at mg L^−1^ levels; suitable for acidic mine waters and industrial effluents	[7,22,102,103,111]

## Data Availability

No new data were created or analyzed in this study. Data sharing is not applicable to this article.

## Abbreviations

The following abbreviations are used in this manuscript:

REEs	Rare Earth Elements
PMs	Precious Metals
HMs	Heavy Metals
WEEE	Waste Electrical and Electronic Equipment
IWW	Industrial Wastewater
